# The Splice Variant of the NCOR2 Gene BQ323636.1 Modulates ACSL4 Expression to Enhance Fatty Acid Metabolism and Support of Tumor Growth in Breast Cancer

**DOI:** 10.3390/ijms26114989

**Published:** 2025-05-22

**Authors:** Ho Tsoi, Chan-Ping You, Koei Ho-Lam Cheung, Yin-Suen Tse, Ui-Soon Khoo

**Affiliations:** Department of Pathology, School of Clinical Medicine, Li Ka Shing Faculty of Medicine, The University of Hong Kong, Hong Kong SAR, China; tsoiho@hku.hk (H.T.); u3006037@connect.hku.hk (C.-P.Y.); cheunghlk@connect.hku.hk (K.H.-L.C.); tyssuen@hku.hk (Y.-S.T.)

**Keywords:** breast cancer, BQ323636.1, ACSL4, lipid metabolism

## Abstract

BQ323636.1 (BQ), a splice variant of NCOR2, is associated with endocrine therapy resistance and poorer prognosis in ER-positive breast cancer. This study investigates the role of BQ in modulating lipid metabolism to support tumor growth. RNA sequencing of BQ-overexpressing breast cancer cells revealed significant enrichment of fatty acid metabolism pathways (hsa01212 and hsa00061; *p* < 0.05), with ACSL4 identified as a key target. We show that BQ disrupts the NCOR2-PPARγ interaction, leading to ACSL4 upregulation, which enhances fatty acid oxidation (FAO), acetyl-CoA by 1.8-fold, and ATP production by 2.5-fold to fuel tumor proliferation. BQ also upregulates FASN and SCD, increasing lipids. A metabolites study with mass spectrometry indicated that BQ overexpression increases the fatty acid amount from 47.97 nmol/10^6^ cells to 75.18 nmol/10^6^ cells in MCF7 and from 56.19 nmol/10^6^ cells to 95.37 nmol/10^6^ cells in ZR-75. BQ activates NRF2, which mitigates ROS-induced stress, promoting cell survival. Targeting ACSL4 with the inhibitor PRGL493 reduced ATP production and suppressed tumor growth in vitro and in vivo, without inducing apoptosis, suggesting a cytostatic effect. PRGL493 treatment can reduce BQ overexpressing tumors by 40% in the xenograft model. These results highlight BQ can serve as a transcriptional hub driving lipid metabolism via ACSL4 in breast cancer. Our findings suggest that ACSL4 inhibition could be a novel therapeutic strategy to overcome treatment resistance in high-BQ expressing ER-positive breast cancer.

## 1. Introduction

Breast cancer is a heterogeneous disease comprised of several molecular subtypes, including Luminal A/B, HER2-enriched, basal-like, and normal-like, each driven by distinct pathogenic mechanisms [1]. Luminal A/B breast cancers are primarily ER-driven [2], whereas HER2-enriched subtypes rely on HER2 signaling, necessitating diverse treatment strategies [3]. However, the development of treatment resistance remains a significant challenge in breast cancer management.

Metabolic reprogramming, a hallmark of cancer, enables tumor cells to meet the high energy and biomass demands required for rapid proliferation, invasion, and metastasis [4]. Due to the particularly high proliferative rate and active nucleotide synthesis of cancer cells, interfering with metabolic pathways has been considered a promising antitumor treatment strategy [5]. Targeting ribonucleotide reductase (RNR) and dihydroorotate dehydrogenase (DHODH) can block nucleotide synthetases, indicating a promising therapeutic strategy. For example, the United States Food and Drug Administration (FDA) has approved inhibiting RNR with hydroxyurea for different cancer types [6]. In breast cancer, particularly ER-positive subtypes, cancer cells rewire their metabolism to favor pathways such as glycolysis, fatty acid oxidation (FAO), and glutamine metabolism, which provide ATP, biosynthetic intermediates, and redox balance [4,7]. The mitochondrial metabolite, α-ketoglutarate (α-KG), was reported to promote colon cancer differentiation by repressing the Wnt signaling pathway [8]. Therefore, energy metabolism pathways, including glycolysis, fatty acid metabolism, glutamine metabolism, the tricarboxylic acid (TCA) cycle, and oxidative phosphorylation (OXPHOS), have emerged as promising metabolic targets.

Lipids comprise thousands of molecules, including fatty acids, triglycerides, sphingolipids, cholesterol, and cholesteryl derivatives. Lipids are widely distributed in cellular organelles and are critical components of all membranes. In addition to their role as structural components, lipids can function as second messengers to transduce signals within cells and serve as important energy sources through β-oxidation of fatty acids [9,10]. Mammalian cells acquire lipids through two mechanisms, i.e., de novo synthesis and uptake. Sterol regulatory element-binding proteins (SREBPs) and peroxisome proliferator-activated receptor-γ (PPARγ) are key transcription factors that regulate the expression of genes involved in lipid synthesis and uptake and play a central role in fatty acid metabolism [11,12]. Fatty acid is degraded via β-oxidation to produce energy in terms of ATP. Five acyl-CoA synthetase long-chain family members (ACSLs; ACSL1 and ACSL3-6) are responsible for catalyzing diverse long-chain fatty acids (LCFAs) into LCFA-acyl-coenzyme A (LCFA-CoA) [13,14]. ACSL1, ACSL3, and ACSL4 have been reported to promote tumor proliferation and sustain stemness by potentiating fatty acid oxidation (FAO) [15,16,17]. ACSL3 and ACSL4 have been shown to enhance the metastatic potential of colorectal carcinoma and breast cancer through increased FAO [18,19]. LCFA-CoA is transported to the mitochondria and broken down to acetyl-CoA through a series of enzymatic reactions. Finally, acetyl-CoA enters the TCA cycle to produce GTP and NADH, which eventually involve ATP production via OXPHOS.

Highly specific inhibitors for FAO are currently unavailable. However, important enzymes involved in fatty acyl-carnitine production, such as carnitine palmitoyltransferase 1A, and fatty acid synthesis and desaturation, including ATP-citrate lyase/ACL, acetyl-CoA carboxylase/ACC, fatty acid synthase/FASN, and stearoyl-CoA desaturase 1/SCD, are targetable. They are being actively studied [20,21,22]. Furthermore, inhibitors of glutamine transporters and glutaminases have been intensively investigated in preclinical and clinical models [23].

BQ323636.1 (BQ), a splice variant of NCOR2, is associated with endocrine resistance and poorer survival in ER-positive breast cancer [24,25]. Our previous studies demonstrated that BQ overexpression confers resistance to multiple anti-endocrine drugs, including tamoxifen and anastrozole, through various mechanisms [26,27,28]. Mechanistically, NCOR2 typically forms a repressor complex with co-regulators to suppress transcription, but BQ, a truncated 362-amino-acid N-terminal variant, disrupts this function by binding to NCOR2 and preventing co-regulator recruitment [24,29]. This interference alters the regulation of transcription factors, [26,27,28,30,31], supporting that BQ could serve as a potential hub for reprogramming breast cancer cell behavior.

Through RNA sequencing, we identified differentially expressed genes and thus confirmed that fatty acid metabolism was significantly enriched. Functional assays confirmed that BQ overexpression could enhance fatty acid levels, acetyl-CoA, and ATP levels. ACSL4 was found to be modulated by BQ through genes that are related to fatty acid metabolism. Mechanistically, the presence of BQ disrupted the interaction between NCOR2 and PPARγ. ChIP assays confirmed that PPARγ bound to the promoter of ACSL4. Targeting ACSL4 compromised the effect of BQ in modulating ATP production, thus suppressing tumor growth. Our study highlights the role of BQ in modulating tumor growth in breast cancer. Our findings suggest that ACSL4 targeting would be a novel approach to suppress breast cancer.

## 2. Results

### 2.1. BQ Overexpression Enhanced Fatty Acid Metabolism to Promote Cancer Cell Proliferation

**Figure 1:**
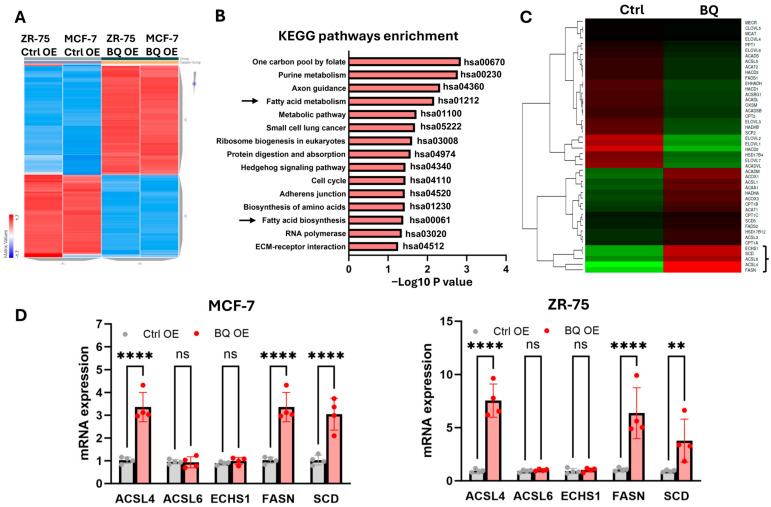
BQ overexpression modulated fatty acid metabolism. (**A**) Heat map showed the differential gene expression between the control (Ctrl OE) and BQ overexpression (BQ OE). A total of 5000 differentially expressed genes were shown. (**B**) Pathway enrichment analysis was performed. Differentially expressed genes were analyzed using the KEGG database, assessed on 26 March 2024, and the top 15 upregulated pathways were shown. Arrows indicate pathways related to fatty acid metabolism. (**C**) Differentially expressed genes related to fatty acid metabolism were analyzed. Genes involved in fatty acid metabolism (hsa01212) and fatty acid biosynthesis (hsa00061) were retrieved from RNA sequencing results and shown in the heatmap. ACSL4, ACSL6, ECHS1, FASN, and SCD, indicated in a bracket, were the most upregulated candidate genes. (**D**) qPCR was employed to validate the findings from RNA sequencing. RT-qPCR was performed. The relative expression of each candidate was determined using ΔΔCT method with actin as the internal control. Results were shown as mean ± SD from 4 independent experiments. **, ****, and ns represented *p* < 0.01, *p* < 0.0001, and non-significant, respectively.

RNA sequencing of BQ-overexpressing ZR-75 and MCF-7 cells revealed differentially expressed genes, and the 5000 differentially expressed genes were shown in the heatmap (Figure 1A, Figure S1A, and Table S1). KEGG pathway enrichment analysis identified the top 15 upregulated pathways, with nearly half related to metabolism, including fatty acid metabolism (hsa01212) and fatty acid biosynthesis (hsa00061) (Figure 1B). Among the upregulated genes in these pathways, ACSL4, ACSL6, ECHS1, FASN, and SCD were the most significant (Figure 1C). ACSL4 and ACSL6 conjugates coenzyme A to fatty acid, forming acyl-coenzyme A, which is a substrate for β-oxidation [13]. ECHS1 catalyzes the hydration of 2-trans-enoyl-CoA intermediates to L-3-hydroxyacyl-CoAs, which is an intermediate step in β-oxidation [32]. FASN plays a significant role in the de novo synthesis of fatty acids [33]. SCD is involved in introducing a double bond into saturated fatty acids, converting them into monounsaturated fatty acids [34]. RT-qPCR validated the upregulation of ACSL4, FASN, and SCD in BQ-overexpressing cells (Figure 1D). These findings led us to hypothesize that BQ overexpression may enhance fatty acid metabolism to sustain the high demand for energy in cancer cells. The energy produced is likely via β-oxidation through increasing the availability of acyl-coenzyme A.

### 2.2. BQ Overexpression Modulated Fatty Acid Profiles in Breast Cancer Cells

**Figure 2:**
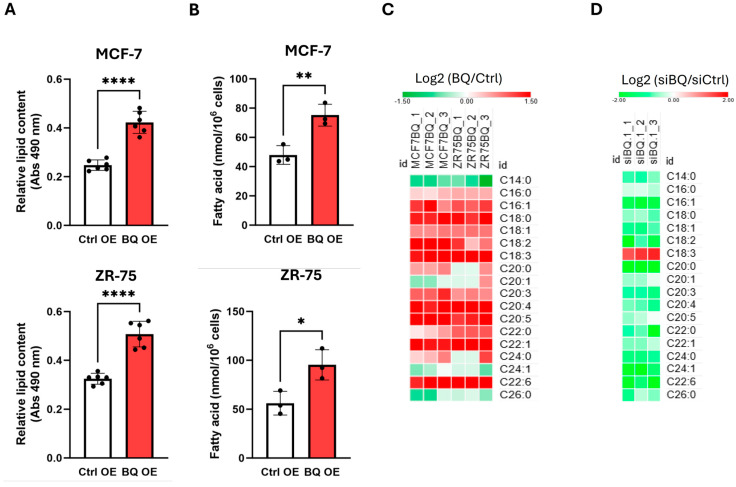
BQ overexpression could modulate the profile of fatty acids. (**A**) Overexpression of BQ enhanced the lipid content in breast cancer cells. Oil Red O solution was employed to stain the lipid, and an absorbance at 490 nm was recorded. Results were shown as mean ± SD from 6 independent experiments. Student’s *t*-test was employed. (**B**) BQ overexpression increased the amount of fatty acids. Fatty acids were quantified using mass spectrometry. Results were shown as mean ± SD from 3 biological replicates. Student’s *t*-test was employed. (**C**) The effect of BQ overexpression on the amounts of saturated, monounsaturated, and polyunsaturated fatty acids (refer to Table S2 for details) was determined through mass spectrometry. The stable BQ overexpressing cells were compared to the control cells. Three biological replicates were shown in the heat map. (**D**) The effect of BQ knockdown on the amounts of each fatty acid was determined. LCC2 cells were used. BQ was downregulated by the siRNA against BQ (siBQ.1) (Figure S1C). After 72 h of the transfection, samples were harvested for mass spectrometry (Table S3). A total of 3 biological replicates were shown. *, **, and **** represented *p* < 0.05, *p* < 0.01, *p* < 0.0001, respectively.

As measured by Oil Red O staining, BQ overexpression significantly increased lipid content in MCF-7 and ZR-75 cells (Figure 2A). Mass spectrometry confirmed elevated total fatty acid levels (Figure 2B and Table S2), with increases in saturated, monounsaturated, and polyunsaturated fatty acids (PUFAs) (Figure 2C and Table S3). Conversely, BQ knockdown in LCC2 cells, which have high endogenous BQ expression (Figure S1B), reduced fatty acid levels (Figure 2D and Figure S1C), confirming BQ’s role in modulating fatty acid profiles.

### 2.3. BQ Overexpression Employed ACSL4 to Enhance ATP Production via FAO

**Figure 3:**
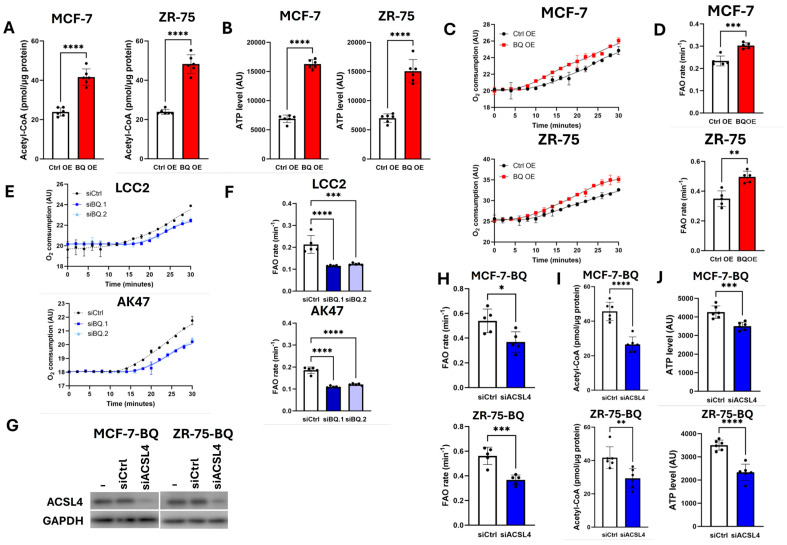
BQ overexpression employed ACSL4 to enhance FAO. (**A**) BQ overexpression increased the amount of acetyl-CoA. Results were shown as mean ± SD from 6 independent experiments. Student’s *t*-test was performed. (**B**) BQ overexpression increased the level of ATP. Results were shown as mean ± SD from 6 independent experiments. Student’s *t*-test was performed. (**C**) BQ overexpression promoted O_2_ consumption. The rate of total O_2_ consumption was monitored. Results were shown as mean ± SD from 5 independent experiments. (**D**) BQ overexpression enhanced the rate FAO. The rate was derived from Figure 4C. Results were shown as mean ± SD from 5 independent experiments. Student’s *t*-test was performed. Knockdown of BQ decreased (**E**) O_2_ consumption and (**F**) FAO. Results were shown as mean ± SD from 5 independent experiments. One-way ANOVA was performed. (**G**) Knockdown of ACSL4. Western blot was employed to confirm the knockdown efficiency of ACSL4 in MCF-7-BQ and ZR-75-BQ. GAPDH was the loading control. The effect of ACSL4 knockdown on (**H**) FAO, (**I**) acetyl-CoA amount, and (**J**) ATP level in MCF-7-BQ and ZR-75-BQ. Results were shown as mean ± SD from 5 independent experiments. Student’s *t*-test was performed. *, **, ***, and **** represented *p* < 0.05, *p* < 0.01, *p* < 0.001, *p* < 0.0001, respectively.

The overexpression of BQ was found to enhance acetyl-CoA (Figure 3A) and ATP levels (Figure 3B). Next, we confirmed that BQ overexpression could enhance oxygen (O_2_) consumption (Figure 3C), and thus the rate of FAO (Figure 3D). In addition, we examined the effect of BQ knockdown by two independent siRNA on LCC2 and AK47 (Figure S1C), which are two cell lines with high endogenous BQ expression (Figure S1B) [24]. The results show that the knockdown of BQ could reduce O_2_ consumption (Figure 3E) and the rate of FAO (Figure 3F). These results indicate that BQ should modulate the use of O_2_ for sustaining high FAO in breast cancer. However, such an effect was compromised by ACSL4 knockdown (Figure 3G), as revealed by reduced FAO (Figure 3H), acetyl-CoA (Figure 3I), and ATP assay (Figure 3J).

### 2.4. Suppression of ACLS4 Inhibited Tumor Growth

**Figure 4:**
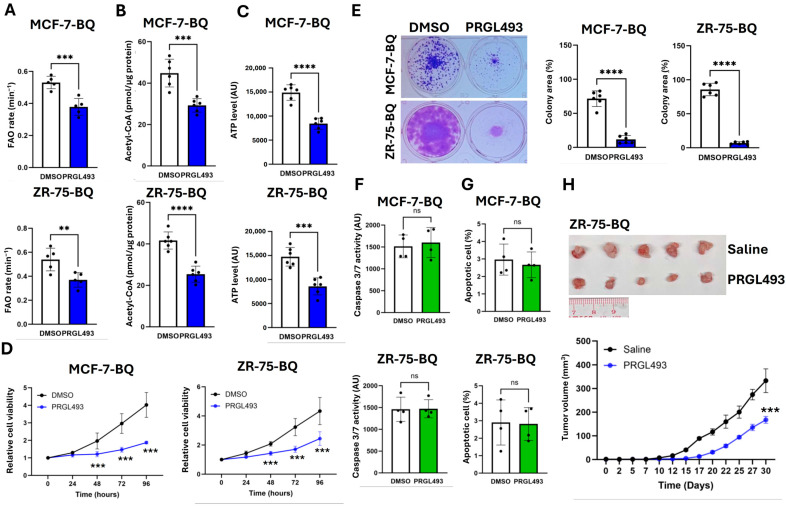
Suppression of ACSL4 inhibited cancer growth mediated by BQ overexpression. ACSL4 inhibition with PRGL493 reduced (**A**) FAO rate, (**B**) acetyl-CoA amount, and (**C**) ATP levels in BQ overexpressing cells. A total of 500 nM of PRGL493 was employed. Assays were performed 48 h posttreatment. Results were shown as mean ± SD from 5 independent experiments. Student’s *t*-test was performed. (**D**) Suppression of ACSL4 repressed cell proliferation. A total of 500 nM of PRGL493 was employed to treat the cells for 96 h. CCK8 was employed to determine cell viability. Results were shown as mean ± SD from 5 independent experiments. Two-way ANOVA was used. (**E**) Colony formation assay. The cells were treated with 500 nM of PRGL493 for 12 days. Representative images were shown, and results were shown as mean ± SD from 6 independent experiments. Inhibition of ACSL4 did not (**F**) activate caspase and (**G**) apoptosis. Results were shown as mean ± SD from 4 independent experiments. Student’s *t*-test was performed. (**H**) Treatment of PRGL493 suppressed tumor growth. Xenografts were established using ZR-75-BQ. 100 µg/Kg of PRGL493 was used. The image showed the tumors after dissection. The tumor growth curve was plotted. Each dot represented one tumor. Results were shown as mean ± SD from 5 independent tumors. Two-way ANOVA was performed. **, ***, ****, and ns represented *p* < 0.01, *p* < 0.001, *p* < 0.0001, and non-significant, respectively.

In addition, a small molecule that inhibited ACSL4, PRGL493, was employed. This compound was identified from docking based virtual screening [35], and the inhibitory effect was demonstrated previously [36]. ACSL4 inhibition with 500 nM of PRGL493, while not affecting ASCL4 and BQ expressions (Figure S2), reduced FAO, acetyl-CoA, and ATP levels in BQ-overexpressing cells (Figure 4A–C), leading to decreased cell proliferation (Figure 4D) and colony formation (Figure 4E). However, ACSL4 inhibition did not activate caspase 3/7 or induce apoptosis (Figure 4F,G and Figure S3), suggesting a cytostatic rather than cytotoxic effect. In vivo, PRGL493 treatment suppressed tumor growth in ZR-75-BQ xenografts (Figure 4H), but did not affect the body weight (Figure S4), highlighting the translational potential of targeting ACSL4 in BQ-high tumors and supporting the safety of PRGL493 at the administrated dose.

### 2.5. BQ Overexpression Disrupted the Interaction Between PPARγ and NCOR2

**Figure 5:**
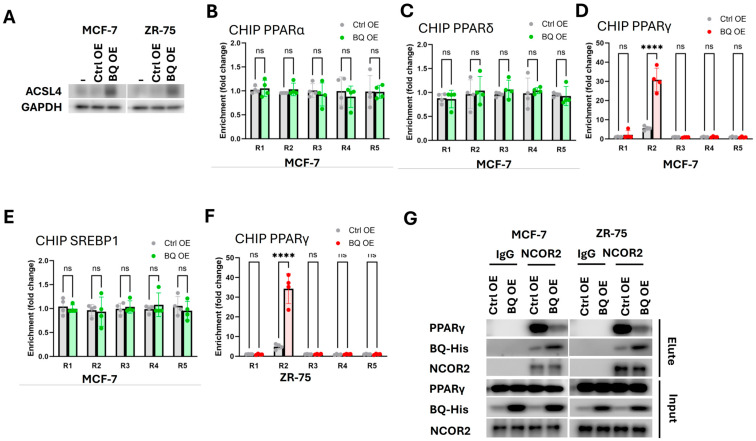
BQ overexpression employed PPARγ to modulate the expression of ACSL4. (**A**) Overexpression of BQ enhances ACSL4 expression. Western blot was performed. GAPDH was the loading control. ChIP with (**B**) anti-PPARα, (**C**) anti-PPARδ, (**D**) anti-PPARγ, and (**E**) anti-SREBP1 were performed on MCF-7 and MCF-7-BQ. A total of 5 regions (R1-R5) within the promoter of ACSL4 (−2000 to +100) were tested. (**F**) Results of ChIP analysis in (**D**) were validated using ZR-75 and ZR-75-BQ. Results were shown as mean ± SD from 4 independent experiments. Two-way ANOVA was used. (**G**) Overexpression of BQ disrupted the interaction between NCOR2 and PPARγ. IP with anti-IgG or anti-NCOR2 was performed. Immunoprecipitant was analyzed for the detection of PPARγ, BQ, and NCOR2. ****, and ns represented *p* < 0.0001, and non-significant, respectively.

We further confirmed that enhanced ACSL4 expression in BQ overexpressing cells (Figure 1D), was also at the protein level (Figure 5A). PPARα, PPARδ, PPARγ, and SREBP1 are transcription factors important in fatty acid metabolism [11,37]. We investigated, by ChIP analysis, whether any of these transcription factors could bind to the promoter of ACSL4 between −2000 to +100 from the transcription starting site. Five pairs of primers were employed. ChIP with anti-PPARα (Figure 5B), anti-PPARδ (Figure 5C), anti-PPARγ (Figure 5D), and anti-SREBP1(Figure 5E) were tested on MCF-7-BQ. Only PPARγ was found to bind to the region 2 (R2) of the ACSL4 promoter (Figure 5D), which was further validated using ZR-75-BQ (Figure 5F). These results confirm that PPARγ can bind to the ACLS4 promoter, with the interaction enhanced when BQ was overexpressed. Next, we showed that NCOR2 could also interact with PPARγ but that BQ overexpression disrupted this interaction (Figure 5G). As NCOR2 is a transcriptional co-repressor gene, this finding suggests that BQ overexpression competes with NCOR2 to diminish the repression of ACSL4, leading to its upregulation, outlining the mechanism through which BQ modulates ACSL4 expression in breast cancer.

### 2.6. BQ Overexpression Employed NRF2 to Overcome High Energy Demand Associated Generation of ROS

**Figure 6:**
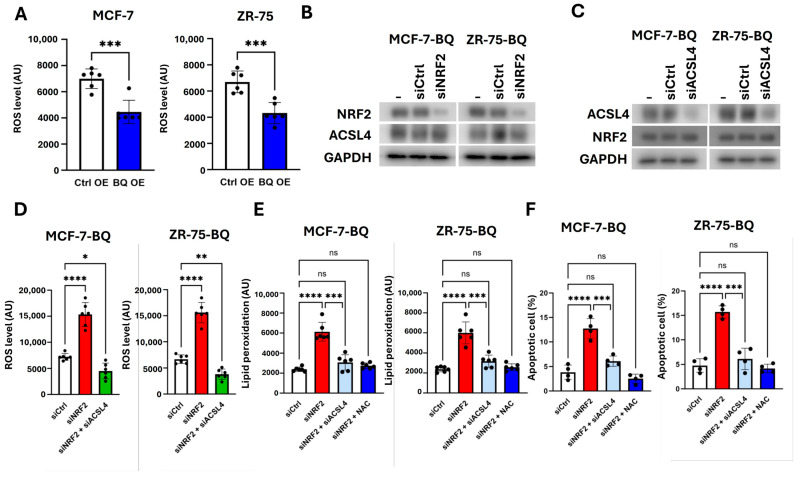
BQ overexpression employed NRF2 to protect cells from ROS-induced apoptosis. (**A**) Overexpression of BQ reduced ROS level. Results were shown as mean ± SD from 6 independent experiments. Student’s *t*-test was employed. (**B**) The effect of NRF2 knockdown on ACSL4. (**C**) The effect of ACSL4 knockdown on NRF2. MCF-7-BQ and ZR-75-BQ were used. GAPDH was the loading control. (**D**) ACLS4 knockdown compromised the effect of NRF2 knockdown on the induction of ROS. The effect of ACSL4 knockdown and of NAC treatment on (**E**) lipid peroxidation and on (**F**) apoptosis mediated by NRF2 knockdown. A total of 5 μM of NAC was employed. Assays were performed 48 h posttreatment. Results were shown as mean ± SD from 6 independent experiments. One-way ANOVA was used. *, **, ***, ****, and ns represented *p* < 0.05, *p* < 0.01, *p* < 0.001, *p* < 0.0001, and non-significant, respectively.

High ATP production is associated with the high production of reactive oxygen species (ROS) as by-products [38]; thus, BQ overexpressing cells are expected to generate high levels of ROS. However, despite increased FAO, BQ-overexpressing cells were observed to exhibit lower ROS levels (Figure 6A). This is likely due to NRF2 upregulation [28]. As NRF2 is a master regulator of anti-oxidants [39], we hypothesized that NRF2 might compromise ROS generated in BQ overexpressing cells. We first confirmed that NRF2 knockdown did not affect ACSL4 expression (Figure 6B) in BQ overexpressing cells. Likewise, ACSL4 knockdown did not affect NRF2 expression (Figure 6C). However, the knockdown of NRF2 significantly enhanced ROS level, which could be reduced when ACSL4 was knocked down together. (Figure 6D). These results suggest that ACSL4 is a key player in ROS production in BQ overexpressing cells. We further confirmed that the knockdown of NRF2 enhanced the degree of lipid peroxidation, as revealed by TBAS assay (Figure 6E). Likewise, NRF2 enhanced lipid peroxidation, which ACSL4 knockdown reversed (Figure 6E). Similar results were seen when a ROS-scavenger NAC (N-Acetyl-L-cysteine) was applied on the NRF2 knockdown cells (Figure 6E). These findings suggest that NRF2 exhibits a protective role though reducing ROS generated by enhanced fatty acid metabolism mediated by BQ overexpression. As expected, the knockdown of NRF2 induced apoptosis in BQ overexpressing cells, compared to the control cells (Figure 6F). Such an effect could be combated by either ACSL4 knockdown or NAC treatment (Figure 6F). Our results illustrated that BQ employed NRF2 to combat ROS production associated with elevated fatty acid metabolism mediated by ACSL4.

## 3. Discussion

Metabolic reprogramming is one of the hallmarks of cancer. It fundamentally supports tumor initiation, progression, and therapeutic resistance by enabling cancer cells to adapt to diverse environmental and therapeutic challenges [4]. In breast cancer, particularly ER-positive subtypes, cancer cells rewire their metabolism to favor pathways like glycolysis, fatty acid oxidation (FAO), and glutamine metabolism, which collectively provide ATP, biosynthetic intermediates, and redox balance to fuel rapid proliferation [7]. This metabolic plasticity is crucial for cancer development, as it allows tumor cells to meet the high energy and biomass demands of uncontrolled growth. For instance, enhanced FAO, as seen in our study, generates acetyl-CoA and ATP, which support nucleotide synthesis and membrane production—key processes for cell division and tumor expansion [10]. Moreover, metabolic reprogramming contributes to treatment failure by enabling cancer cells to survive therapeutic stress. Increased FAO and glycolysis can provide ATP to fuel drug efflux pumps, such as P-glycoprotein, thereby reducing intracellular drug concentrations and conferring resistance to chemotherapies like doxorubicin [1]. Additionally, an altered metabolism supports redox homeostasis by producing NADPH and antioxidants, counteracting the oxidative stress induced by therapies that rely on ROS for cytotoxicity [38]. Metabolic shifts also play a pivotal role in cancer metastasis, a major cause of mortality in breast cancer. An enhanced FAO provides energy for epithelial-mesenchymal transition (EMT), migration, and invasion, while lipid synthesis supports membrane remodeling necessary for metastatic colonization [19]. Furthermore, metastatic cells often upregulate glutamine metabolism to fuel the TCA cycle, ensuring energy production in nutrient-scarce metastatic sites [7]. These metabolic adaptations collectively enable breast cancer cells to thrive under diverse conditions, underscoring the need to target metabolic pathways for effective therapeutic strategies. Our study findings support BQ323636.1 (BQ), a splice variant of NCOR2, as a key orchestrator of such metabolic reprogramming, driving lipid metabolism via ACSL4 to support tumor growth, survival, and resistance in ER-positive breast cancer.

We demonstrate that BQ disrupts the repressive function of NCOR2 by interfering with its interaction with PPARγ, leading to the upregulation of ACSL4 (Figure 5). This enhances β-oxidation, increasing acetyl-CoA and ATP production to fuel tumor proliferation, as evidenced by elevated levels of these metabolites in BQ-overexpressing MCF-7 and ZR-75 cells (Figure 3A,B), leading to increased oxygen consumption and FAO rates (Figure 3C,D). The knockdown of BQ reversed these findings (Figure 3E,F). Similar findings were obtained on ACSL4 inhibition with PRGL493, which confirms to role of BQ and ASCL4 in energy production (Figure 4A–C). These findings highlight how BQ exploits FAO to meet the energy demands of cancer cells, contributing to the metabolic adaptations that drive tumor development.

The upregulation of FASN and SCD by BQ (Figure 1D) reveals a broader lipid metabolism network. FASN drives de novo fatty acid synthesis, increasing lipid levels, as also observed in our BQ overexpressing cells (Figure 2A). SCD enhances monounsaturated and polyunsaturated fatty acids (PUFAs), as also evidenced with BQ overexpression (Figure 2B,C), potentially aiding membrane fluidity and β-oxidation efficiency [19]. The primary role of ACSL4 is to promote energy production via β-oxidation through the generation of substrates for entry into this process, rather than lipid synthesis, making it a specific target for disrupting tumor metabolism. The interplay between ACSL4, FASN, and SCD underscores a coordinated lipid metabolism axis in BQ-driven breast cancer, with ACSL4 as the key effector of energy production, potentially supporting metastatic potential by providing energy for EMT and invasion.

We have previously shown that BQ can regulate the NRF2 signaling pathway via interference with NCOR2 suppressive activity [28]. The modulation of NRF2 by BQ provides a survival advantage under metabolic stress, further contributing to treatment failure. Despite elevated β-oxidation, which typically increases reactive oxygen species (ROS) [38], BQ-overexpressing cells exhibit lower ROS levels due to NRF2 upregulation (Figure 6A). NRF2, a master antioxidant regulator [39], mitigates ROS from ACSL4-mediated metabolism, as evidenced by increased ROS on NRF2-knockdown (Figure 6D). It thus prevents oxidative damage and apoptosis (Figure 6E,F). The mitigation of apoptosis by the ROS scavenger, NAC, in NRF2-knockdown cells (Figure 6F) confirms ROS as the driver of cell death in this context. This protective mechanism likely enhances tumor survival, particularly in the presence of therapies like doxorubicin, which rely on oxidative stress to induce cytotoxicity [1]. By counteracting ROS, NRF2 may enable BQ-overexpressing cells to resist doxorubicin, in alignment with the role of metabolic reprogramming in treatment failure.

The genetic signaling pathway initiated by BQ overexpression, which disrupts the NCOR2–PPARγ interaction to de-repress ACSL4 (Figure 5), positions BQ as a transcriptional hub in breast cancer. PPARγ, a key regulator of lipid metabolism [11], directly binds to the ACSL4 promoter (Figure 5), and its de-repression by BQ amplifies FAO, fueling tumor growth (Figure 4) and proliferation (Figure 4D,E). This pathway underscores the association of BQ with poorer survival, as previously demonstrated in over 300 ER-positive breast cancer cases [24]. This highlights its role in driving tumor progression, including potential metastatic behavior which is known to be driven by enhanced lipid metabolism [40,41].

From a translational perspective, targeting ACSL4 offers potential promise for ER-positive breast cancer treatment, particularly in high-BQ expressing patients. ACSL4 inhibition reduces energy production and tumor growth without inducing apoptosis (Figure 4), suggesting a cytostatic effect that could be leveraged for combination therapies. Notably, our prior studies indicate that BQ overexpression confers epirubicin resistance [28], a major challenge in ER-positive breast cancer treatment [1]. ACSL4 inhibition may resensitize BQ-overexpressing tumors to epirubicin by depleting ATP, thereby impairing the energy-dependent survival mechanisms that counteract chemotherapy-induced stress. This approach could improve outcomes for patients with high-BQ expression, who generally have poorer prognosis and resistance to standard therapies like doxorubicin and tamoxifen [24]. ACSL4 inhibitors could be explored as adjuvant, given in combination with existing treatments, offering a novel strategy to overcome resistance in this subset of patients. Given the lack of highly specific FAO inhibitors [10], developing targeted ACSL4 inhibitors represents a critical step forward. Preclinical models testing ACSL4 inhibitors in combination with doxorubicin and clinical trials in BQ-high cohorts are warranted to validate this approach. Additionally, integrating BQ expression as a biomarker in clinical diagnostics could guide patient stratification, ensuring that ACSL4-targeted therapies can be applied to those most likely to benefit.

Future studies should further dissect the contributions of FASN and SCD to BQ-driven lipid metabolism, as their roles in substrate availability and membrane dynamics may amplify ACSL4’s effects, particularly in supporting metastasis. Investigating other transcription factors modulated by BQ-NCOR2 disruption could uncover additional metabolic targets. Finally, exploring the broader impact of ACSL4 inhibition on tumor microenvironment interactions, such as immune evasion or stromal support, may reveal additional therapeutic opportunities in ER-positive breast cancer.

## 4. Material and Methods

### 4.1. Cell Culture, Transfection, siRNA, and Chemicals

MCF-7 (HTB-22) and ZR-75-1 (CRL-1500) were obtained from the American Type Culture Collection (Manassas, VA, USA). LCC2 and AK-47 were kindly provided by Dr. Robert Clarke (Georgetown University Medical School, Washington, DC, USA). MCF-7-Ctrl and ZR-75-Ctrl were established by stably transfecting with mammalian expression plasmid pcDNA3.1-His to MCF-7 and ZR-75-1, respectively. MCF-7-BQ and ZR-75-BQ, stably transfected by mammalian expression plasmid pcDNA3.1-His-BQ to MCF-7 and ZR-75-1, respectively, were used [24]. MCF-7, MCF-7-Ctrl, MCF-7-BQ, ZR-75, ZR-75-Ctrl, ZR-75-BQ, LCC2, and AK-47 cells were cultured and maintained in DMEM (12100046, Thermo Fisher Scientific, Waltham, MA, USA) containing 10% FBS (26140079, Thermo Fisher Scientific, Waltham, MA, USA) and 1% P/S (10378016, Thermo Fisher Scientific, Waltham, MA, USA). The cell lines were kept in the incubator at 37 °C and supplied with 5% CO_2_. Small interference RNAs (siRNA) that target NRF2 (sc-37030) and ACSL4 (sc-60619) were purchased from Santa Cruz Biotechnology (Dallas, TX, USA). Non-targeting siRNA (siCtrl; sc-37007) was obtained from Santa Cruz Biotechnology (Dallas, TX, USA). The knockdown of BQ was performed according to our previous study [31]. Oligofectamine™ transfection reagent (12252011, Thermo Fisher Scientific, Waltham, MA, USA) was used. A total of 20 nM of the siRNA was used, and the transfection was performed according to the manufacturer’s manual. PRGL493 (HY-139180, MedChemExpress LLC, Monmouth Junction, NJ, USA; purity >98% as confirmed by HPLC) was purchased. N-Acetyl-L-cysteine (NAC; A7250, Sigma-Aldrich, USA; St. Louis, MO, USA; purity ≥99% as confirmed by HPLC) was used.

### 4.2. RNA Sequencing and Pathway Enrichment Analysis

Sequencing work was performed by the Centre for PanorOmic Sciences (CPOS) at The University of Hong Kong using Illumina NovaSeq 6000 for Pair-End 151bp sequencing. The generation of cDNA libraries and data analyses were performed according to our previous study [42]. Pathway enrichment analysis was performed using data from the Kyoto Encyclopedia of Genes and Genomes (KEGG) [43]. Differential gene expression (heat map) and top scored pathways were shown using BioJupies using default settings [44]. The sequencing results are publicly available at GSE295979.

### 4.3. Targeted Quantitation of C14:0-C26:0 Fatty Acids Analysis

A total of 100 µL of chloroform with 20 µg C19:0 fatty acid internal standard was spiked to the sample. The sample was extracted with 5 rounds of 2:1 Chloroform/Methanol, followed by sonication. After centrifugation, the supernatant was further cleaned by liquid–liquid extraction in 0.73% NaCl and Methanol. The resultant mixture was dried under a fume of N2 at 45 °C before transesterification. A total of 1 mL of methanol and 50 µL of concentrated hydrochloric acid (35%, *w*/*w*) were added to the sample. The solution was overlaid with nitrogen and the tube was tightly closed. After vortexing, the tube was heated at 100 °C for 1.5 h. Once cooled to room temperature, 1 mL of hexane and 1 mL of water were added for FAMEs extraction. The tube was vortexed and after phase separation, up to 1 µL the hexane phase was injected for GC-MS analysis. A GC/MS chromatogram was acquired in SCAN and SIM mode in an Agilent 7890B GC—Agilent 7010 Triple Quadrapole Mass Spectrometer system (Agilent Technologies, Santa Clara, CA, USA). The sample was separated through an Agilent DB-23 capillary column (60 m × 0.25 mm ID, 0.15 µm film thickness) under constant pressure at 33.4 psi. The GC oven program started at 50 °C (hold time 1 min) and was increased to 175 °C at a ramp rate of 25 °C/min. The temperature was then raised to 190 °C (hold time 5 min) at a ramp rate of 3.5 °C/min. Finally, the temperature was raised to 220 °C (hold time 4 min) at a ramp rate of 2 °C/min. The inlet temperature and transfer line temperatures were 250 °C and 280 °C, respectively. Characteristic fragment ions (*m*/*z* 55, 67, 69, 74, 79, 81, 83, 87, 91, 93, 95, 96, 97, 115, 127, and 143) were monitored in SIM mode throughout the run. Mass spectra from *m*/*z* 50–350 were acquired in SCAN mode on 29 December 2021. Data analysis was performed using the Agilent MassHunter Workstation Quantitative Analysis Software (version 11.0; Agilent Technologies, Santa Clara, CA, USA). Linear calibration curves for each analyte were generated by plotting the peak area ratio of external/internal standard against standard concentration at different concentration levels. Analytes were confirmed by comparing the ratio of characteristic fragment ions in the sample and standard. Three biological replicates were employed. The ratios of BQ/Ctrl and siBQ/siCtrl were determined and Log_2_ transformed. The relative amount of each fatty acid is shown in Supplementary Table S1 and in the heatmap. The heatmap was generated using Morpheus (https://software.broadinstitute.org/morpheus, accessed on 26 March 2024), which is established by Broad Institute (Cambridge, MA, USA).

### 4.4. RNA Extraction and RT-qPCR

RNA extraction was performed with TRIzol™ Reagent (15596026, Thermo Fisher Scientific, Waltham, MA, USA) or RNA isolater Total RNA Extraction Reagent (R401-01, Vazyme, Nanjing, China). A total of 0.5 µg of RNA was used for cDNA synthesis using High-Capacity cDNA Reverse Transcription Kit (4368814, Thermo Fisher Scientific, Waltham, MA, USA). qPCR was performed using ChamQ Universal SYBR qPCR Master Mix (Q711-02, Vazyme, Nanjing, China). Primer sequences were shown in Supplementary Table S2. Samples were duplicated. The relative gene expression was determines using the ΔΔCT method with actin as the internal control. The following primes (5′→3′) were used: ACSL4-F, AGC ACT GAA CCT GGG AAA GA; ACSL4-R, TCA GCA ACA GCA AAC AGA CC; ACSL6-F, CCT TGC TGG GGT CTT CTA GT, ACSL6-R, TTC CCA CCT TCT GCC TTG AT; ECHS1-F, CTG CTT CAC ACC TCT GCT TG; ECHS1-R, CGC AGC AAT TGG AGA GGA AC; FASN-F, CGA GGC TGC TAG ATG TAG GT; FASN-R, GCT CTT CAC AGA CCA GGA GT; SCD-F, TGA AAG CCA ACA ACT CTG CC; SCD-R, CCT GGG AGG CAA TAA GGG AA; ACTIN-F, ATG TGC AAG GCC GGT TTC GC; and ACTIN-R, CGA CAC GCA GCT CAT TGT AG.

### 4.5. Chromatin Immunoprecipitation (ChIP)

ChIP was performed using BeyoChIP™ Enzymatic ChIP Assay Kit (P2083S, Beyotime Biotechnology, Haimen, China). The following antibodies were tested: anti-PPARα (66826-1-Ig, Proteintech Group Inc, Rosemont, IL, USA), anti-PPARδ (60193-1-Ig, Proteintech Group Inc, Rosemont, IL, USA), anti-PPARγ, and anti-SREBP1 (14088-1-AP, Proteintech Group Inc, Rosemont, IL, USA). The promoter sequence of ACLS4 from -2000 to +100 was retrieved from The Eukaryotic Promoter Database, assessed on 15 March 2024 [45]. A total of 5 potential binding sites, R1 to R5, at different positions of ACSL4 promoter were studied. The following primers (5′→3′) were used: ACSL4_-1819 to -1730-R1-F, TCT TGT AGA GCT GGA GGA AC; ACSL4_-1819 to -1730-R1-R, TTG TCG GTC TCT TAG CCC AG; ACSL4_-1437 to -1326-R2-F, ACT CGT TGT TTG AAG TAC TGA AC; ACSL4_-1437 to -1326-R2-R, TCC AAG AAA CCC ACC CAC TT; ACSL4_-968 to -851-R3-F, TCA CCA TTA CGC ATC AGG GA; ACSL4_-968 to -851-R3-R, AGG GCT TCA AAA GGG ACA GA; ACSL4_-489 to -384-R4-F, CTT TTG AGG GCA GCT GGA AG; ACSL4_-489 to -384-R4-R, CAG TGA GGC GCA GAA TTG G; ACSL4_-156 to -82-R5-F, CGA TTC GGC TGG CTC TGC; and ACSL4_-156 to -82-R5-R, GAG CCC GGA AAA GAC GC G.

### 4.6. Co-Immunoprecipitation (CoIP)

Binding buffer (20 mM Tris-Cl, pH7.4, 100 mM NaCl, 5 mM MgCl_2_, 0.5% NP-40, 10% Glycerol) was used. A total of 2 × 10^6^ cells were palleted and lyzed in 500 μL of the lysis buffer. A protein assay was performed (5000112, Bio-Rad, Hercules, CA, USA) and cell lysates containing 1 mg of proteins with volume adjusted to 100 μL with binding buffer were subjected to immunoprecipitated with anti-NCOR2 (1:100; #62370, Cell Signaling Technology, Danvers, MA, USA) or anti-IgG (1:100; 30000-0-AP, Proteintech Group Inc, Rosemont, IL, USA) at 4 °C overnight. The immunoprecipitant was incubated with 50 μL of Protein A/G Magnetic Beads (PB101-01, Vazyme, Nanjing, China) for 2 h at 4 °C. The beads were then washed three times with 1 mL of binding buffer for 10 min each. The beads were collected and incubated with 50 μL of 2× SDS sample buffer at 99 °C for 10 min. The samples were then analyzed with SDS-PAGE.

### 4.7. Western Blot

A lysis buffer (20 mM Tris-Cl, pH7.4, 150 mM NaCl, 0.5% NP-40, 0.5% SDS) was prepared. Proteins were extracted using 200 μL of lysis buffer. A protein assay was performed (5000112; Bio-Rad, Hercules, CA, USA). A total of 20 μg of protein were analyzed with SDS-PAGE. The following primary antibodies were used: anti-ACSL4 (1:400; sc-365230, Santa Cruz Biotechnology, Dallas, TX, USA), anti-BQ (1:1000; D-12, Veritech Ltd., Hong Kong, China), anti-GAPDH (1:5000; sc-365062, Santa Cruz Biotechnology, Dallas, TX, USA), anti-cleaved caspase 3 (1:1000; 25128-1-AP, Proteintech Group Inc, Rosemont, IL, USA), anti-HSP90 (1:4000; #4874, Cell Signaling Technology, Danvers, MA, USA), anti-NCOR2 (1:1000; #62370, Cell Signaling Technology, Danvers, MA, USA), anti-NRF2 (1:1000; #12721, Cell Signaling Technology, Danvers, MA, USA), anti-PARP1 (1:1000; #9542, Cell Signaling Technology, Danvers, MA, USA), and anti-PPARγ (1:2000; 66936-1-Ig, Proteintech Group Inc, Rosemont, IL, USA). The following secondary antibodies were used: anti-mouse HRP (1:5000; P0447, Agilent Dako, Santa Clara, CA, USA) and anti-rabbit HRP (1:5000; P0260, Agilent Dako, Santa Clara, CA, USA). The signal was developed using BeyoECL Moon (P0018FS, Beyotime Biotechnology, Haimen, China) or BeyoECL Star (P0018AS, Beyotime Biotechnology, Haimen, China). Amersham Imager 680 (GE HealthCare, Chicago, IL, USA) was used to record the signal.

### 4.8. Lipid Content Measurement

The steps were performed according to a previous report [46]. In brief, Oil Red O stock solution was prepared by dissolving 0.7 g of Oil Red O (O0625; Sigma-Aldrich, USA; St. Louis, MO, USA) in 200 mL of isopropanol. Oil Red O stock solution was prepared by mixing 60 mL of Oil Red O stock solution with 40 mL of ddH_2_O. A total of 1 × 10^6^ cells were fixed using 250 μL formalin 10% (*v*/*v*) in PBS. The cells were washed with 250 μL 60% (*v*/*v*) isopropanol. After drying, 150 μL of Oil Red O working solution were added per well onto the cells. The cells were incubated at room temperature for 10 min. The cells were washed with running distilled water five times. The plate was dried in a fume hood overnight. A total of 500 μL isopropanol was added and incubated for 15 min. The solution was transferred to a 96-well plate in triplicates at 100 μL each. An absorbance at 490 nm was recorded using microplate reader Infinite F200 (Tecan, Seestrasse, Switzerland). Relative lipid content was determined by comparing to untreated control.

### 4.9. Functional Assays

Acetyl-CoA Assay Kit (NBP3-24455; Novus Biologicals, Centennial, CO, USA) was used. Cells in a 24-well plate were lyzed by 200 μL of lysis buffer (20 mM Tris-Cl pH7.4, 100 nM NaCl, 0.5% NP-40, 0.1% SDS). Protein concentration was determined using DC protein assay (5000112; Bio-Rad, Hercules, CA, USA). A total of 1 μg of protein was used in the assay. ATP Determination Kit (A22066; Thermo Fisher Scientific, Waltham, MA, USA) was used. Chemiluminescence was recorded by microplate reader Infinite F200 (Tecan, Seestrasse, Switzerland). Fatty Acid Oxidation Complete Assay (ab222944; Abcam, Cambridge, UK) was used. Fluorescence signal was monitored using SpectraMax^®^ i3x Multi-Mode Microplate Reader (Molecular Devices, San Jose, CA, USA). FAO was equal to the total O_2_ consumption minus O_2_ consumption in the presence of CPT1a inhibitor etomoxir which blocked Fatty acid-CoA from being imported to the mitochondria. The inhibitor was supplied in the assay kit. Cell viability was determined using cell countering kit-8 (CCK8; C0038, Beyotime, China). The colony formation assay was performed on a 12-well plate. A total of 5000 cells were seeded. Colonies were visualized by staining with 0.05% of crystal violet (61135; Sigma-Aldrich, USA; St. Louis, MO, USA). Images were captured using a digital camera and the image was analyzed with Image J to determine the area (%) with colonies. Caspase activity was determined using Caspase-Glo^®^ 3/7 Assay System (G8090; Promega, Madison, WI, USA). A total of 20,000 cells were seeded in a 96-well plate and chemiluminescence was recorded by microplate reader Infinite F200 (Tecan, Seestrasse, Switzerland). Apoptosis was evaluated using Dead Cell Apoptosis Kits with Annexin V (V13245; Thermo Fisher Scientific, Waltham, MA, USA). Flow cytometry was employed to analyze the cells. LSRFortessa™ (BD Biosciences, Franklin Lakes, NJ, USA) was used.

### 4.10. Xenograft

The procedure was approved by the HKU Committee on the Use of Live Animals in Teaching and Research (CULATR 5103-19). Six-week-old female nude mice (strain: BALB/cAnN-nu) were used. A total of 1 × 10^7^ cells of ZR-75-BQ were trypsinized and mixed with Matrigel (Corning; 356234; BD Bioscience, Franklin Lakes, NJ, USA) at 1:1 ratio in volume. The cell mixture was implanted into the mammary fat pad. A caliper was used to measure the size of the tumor. The volume of the subcutaneously xenografted tumors was determined by current standard technique [27]. Saline or PRGL493 (100 µg/Kg; HY-139180, MedChemExpress LLC, Monmouth Junction, NJ, USA) was administrated through IP injection twice per week. At the endpoint of the experiment, mice were euthanized and tumors were harvested.

### 4.11. Statistical Analysis

All numerical results were stored and handled in Excel (Microsoft). GraphPad Prism 10 was used to perform statistical analyses and export graphs. One-way ANOVA and two-way ANOVA with Bonferroni’s posttest were used for comparing the statistical difference among multiple groups. Student’s *t*-test was used to compare the statistical difference between two groups. Before applying parametric tests, normality was assessed for each experimental group using the Shapiro–Wilk test (*p* > 0.05 for all groups) or visual inspection of quantile-quantile plots in GraphPad Prism. These results confirmed that the data met the normality assumption, supporting the use of parametric tests such as Student’s *t*-test and ANOVA. *, **, ***, ****, and ns represented *p* < 0.05, *p* < 0.01, *p* < 0.001, *p* < 0.0001, and non-significant, respectively.

## 5. Conclusions

This study demonstrates that BQ323636.1 (BQ) drives lipid metabolism in ER-positive breast cancer by upregulating ACSL4 through disruption of the NCOR2-PPARγ interaction, enhancing FAO to fuel tumor growth. BQ also modulates FASN and SCD to support lipid availability and employs NRF2 to mitigate ROS-induced stress, promoting cell survival and contributing to treatment resistance. Targeting ACSL4 with inhibitors like PRGL493 suppresses tumor growth by reducing energy production, offering a promising therapeutic strategy for BQ-high patients. These findings underscore BQ’s role as a metabolic hub in breast cancer and highlight ACSL4 as a novel target to overcome resistance in ER-positive breast cancer.

## Data Availability

The materials and resources in this study are available from the corresponding author upon reasonable request.

## Supplementary Materials

The following supporting information can be downloaded at: https://www.mdpi.com/article/10.3390/ijms26114989/s1.
