# Supplementary material for: The Splice Variant of the NCOR2 Gene BQ323636.1 Modulates ACSL4 Expression to Enhance Fatty Acid Metabolism and Support of Tumor Growth in Breast Cancer
Source: Int J Mol Sci. 2025 May 22;26(11):4989. doi: 10.3390/ijms26114989 (PMC12154026; doi:10.3390/ijms26114989)
Supplement: Supplementary file 1 [file ijms-26-04989-s001.zip › 20250516_clean_ACSL4_supplementary figure legends.pdf]

Supplementary figure legends

Figure S1 BQ expression in different conditions. (A) Expression of BQ in the stable control (Ctrl OE) and BQ overexpression (BQ OE) cell lines. Western blot was performed with GAPDH as the loading control. (B) Comparing endogenous BQ expression among 4 breast cancer cell lines. Western blot was used. GAPDH was used as the loading control. (C) Knockdown of BQ. Western blot was employed to confirm the knockdown efficiency of BQ in LCC2 and AK47. 20 nM of the siRNA was used. GAPDH was the loading control.

Figure S2 ACSL4 inhibitor PRGL493 did not affect ACSL4 and BQ expressions. MCF-7-BQ and ZR-75-BQ were treated with 500 nM of PRGL493. Cells were harvested after 48 hours of the treatment. Western blot was performed. HSP90 was used as the loading control.

Figure S3 ACSL4 knockdown and inhibition had no effect on caspase activation and PARP1 cleavage. MCF-7-BQ and ZR-75-BQ were treated with 20 nM of the siRNA or 500 nM of PRGL493. Western blot was performed 48 hour posttreatment. GAPDH was the loading control.

Figure S4 The effect of 100 μg/Kg of PRGL493 administration on body weight of nude weight. Results were shown as mean ± SD of 5 mice.
