# Supplementary figures and images for: The Splice Variant of the NCOR2 Gene BQ323636.1 Modulates ACSL4 Expression to Enhance Fatty Acid Metabolism and Support of Tumor Growth in Breast Cancer
Source: Int J Mol Sci. 2025 May 22;26(11):4989. doi: 10.3390/ijms26114989 (PMC12154026; doi:10.3390/ijms26114989)

Figure 3G

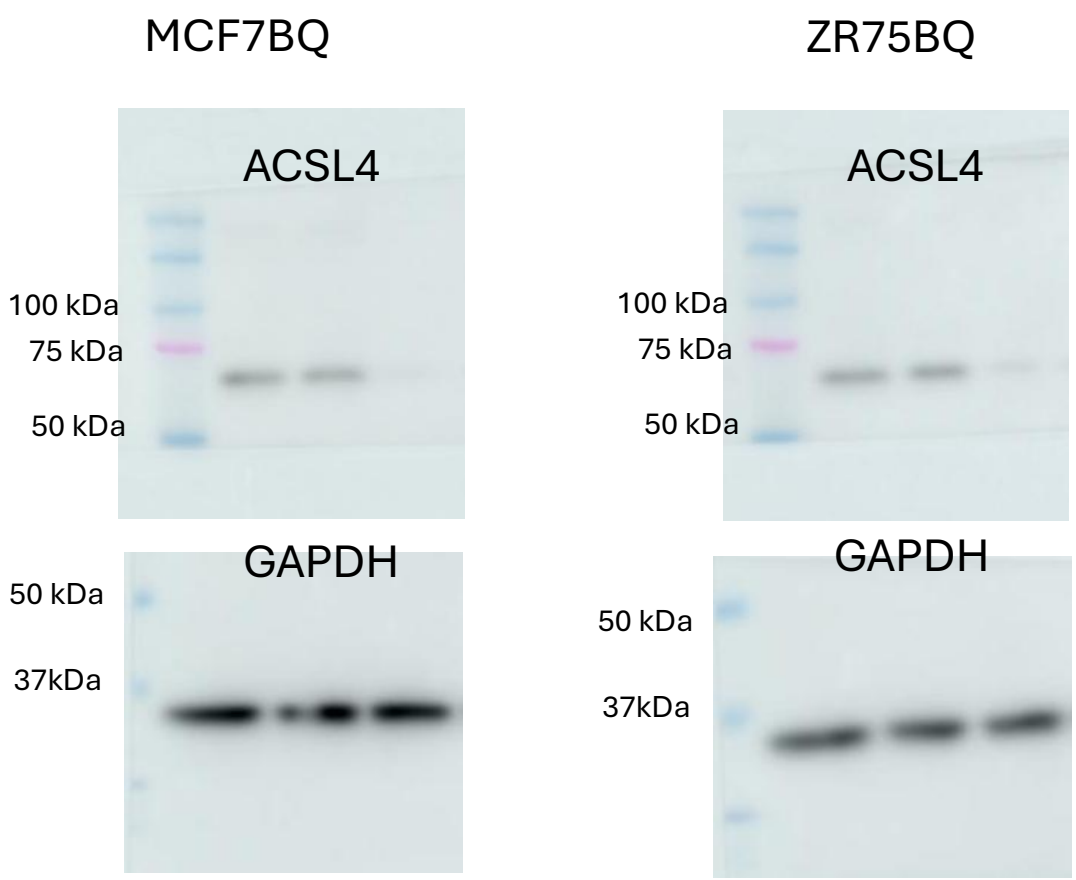

Figure 5G

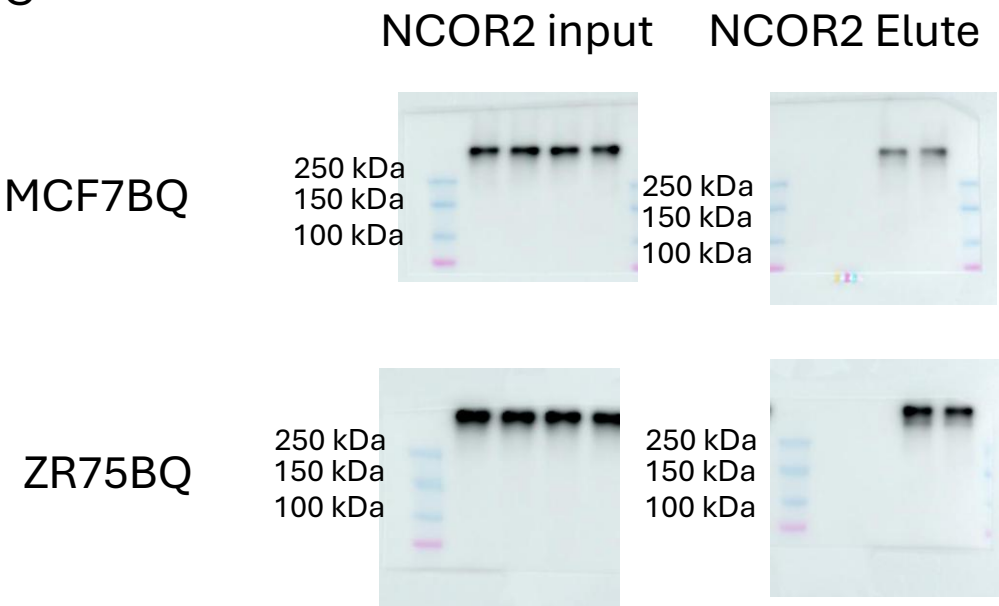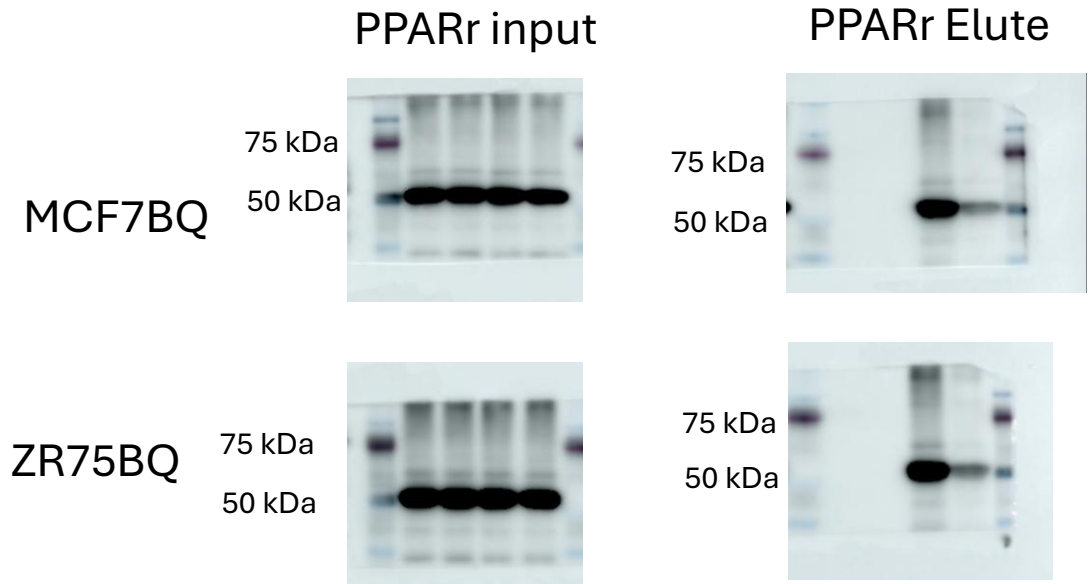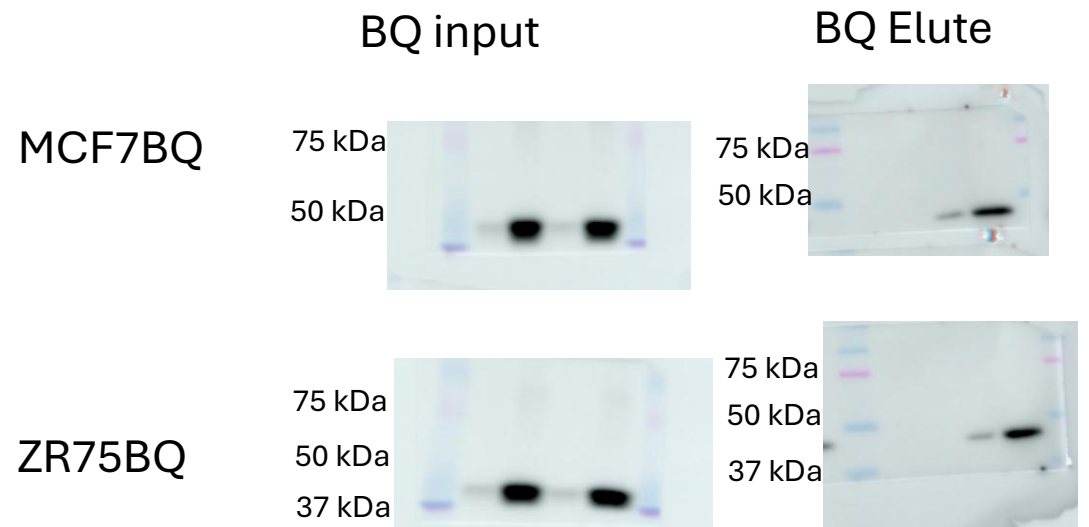

Figure 6B

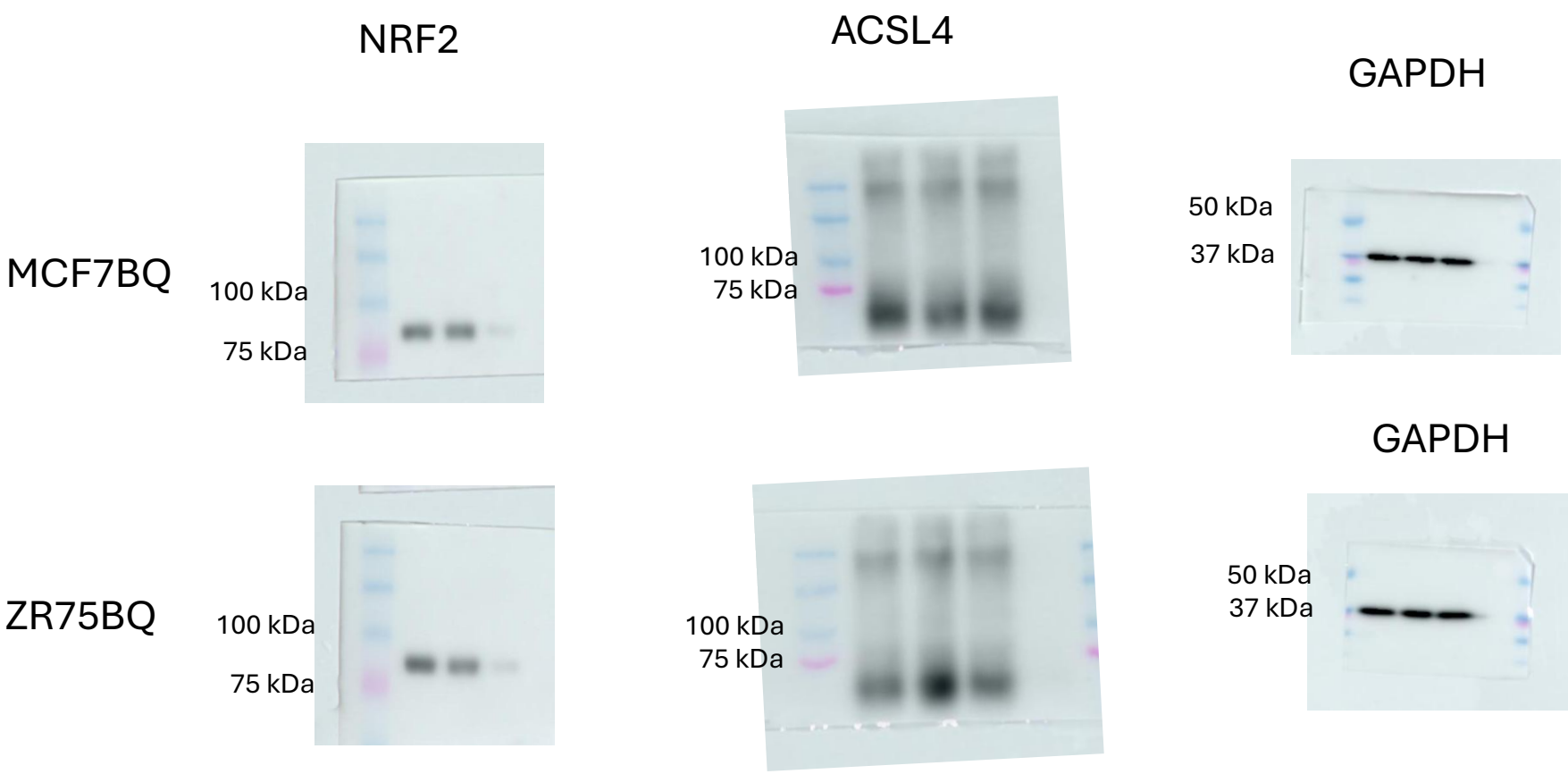

Figure 6C

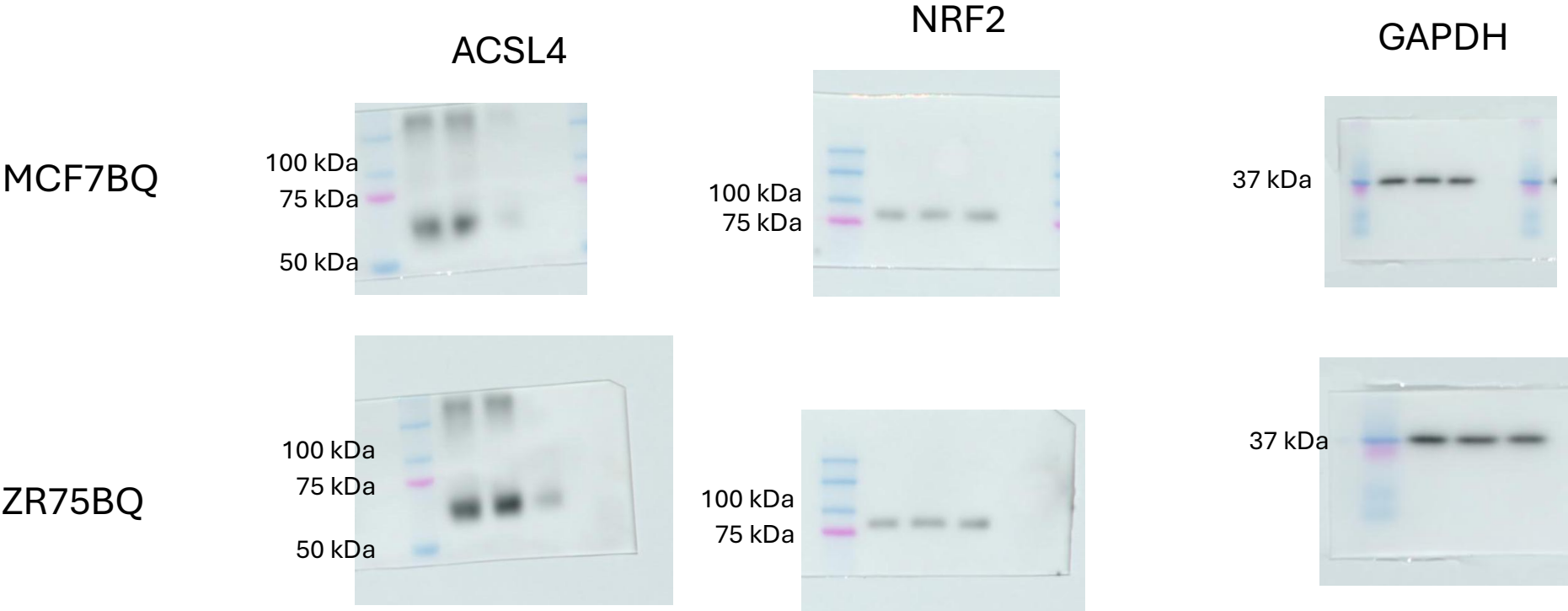

Figure S1A

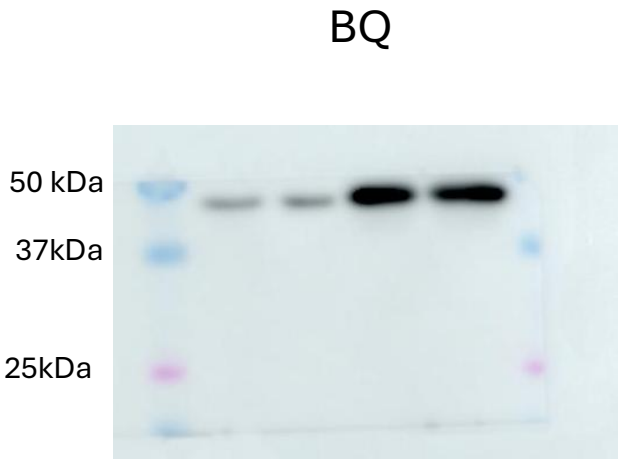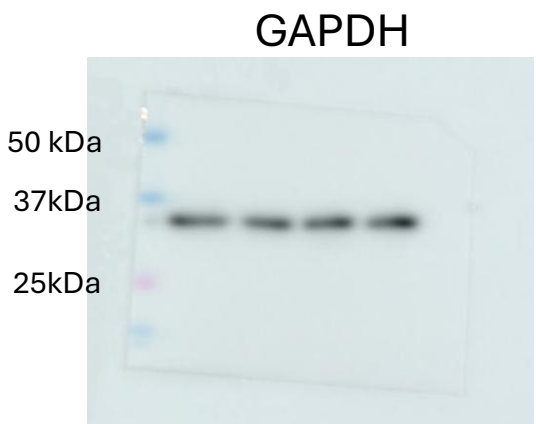

Figure S1B

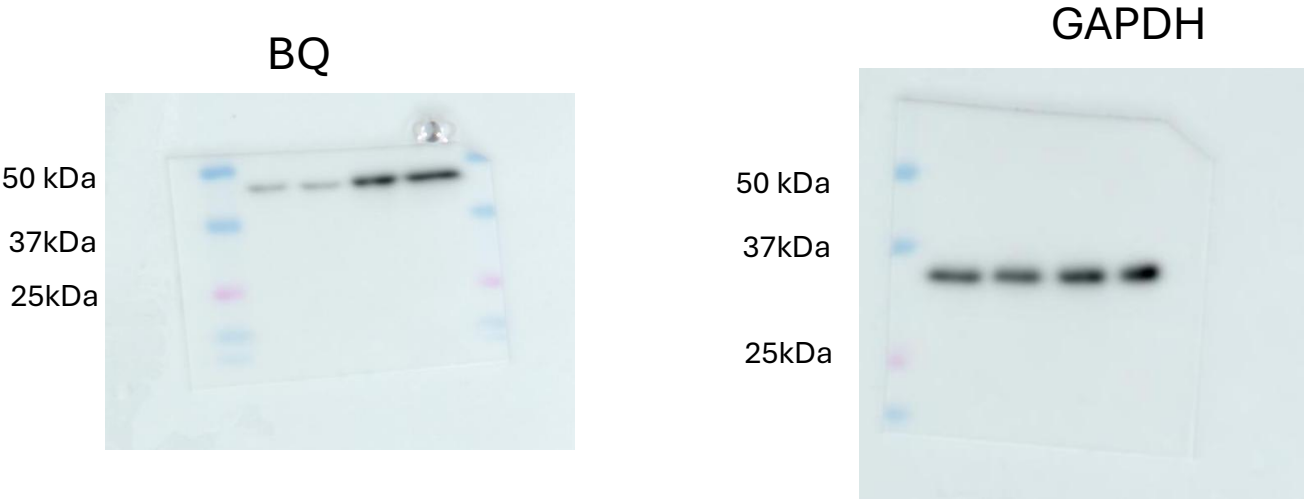

Figure S1C

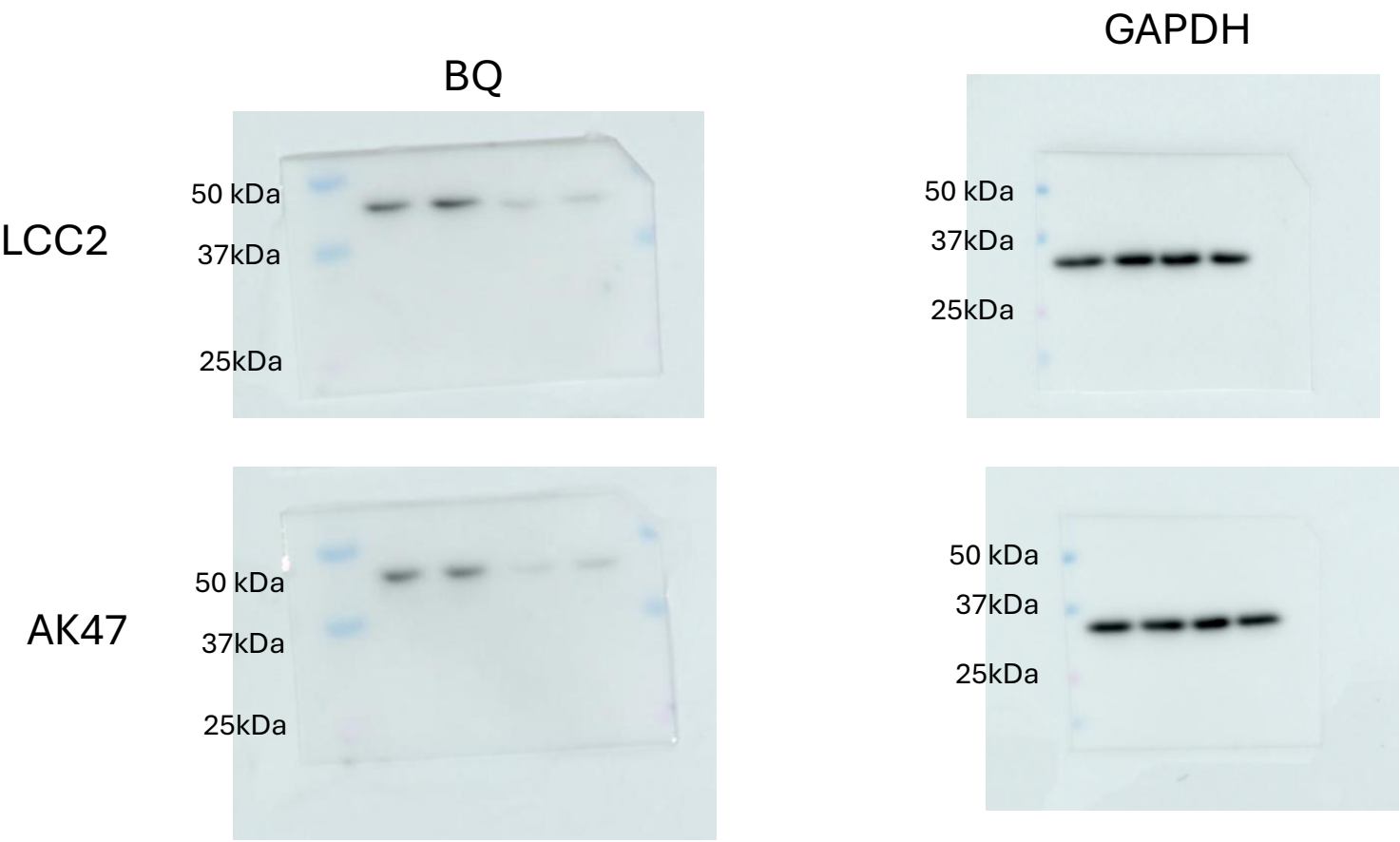

Figure S2

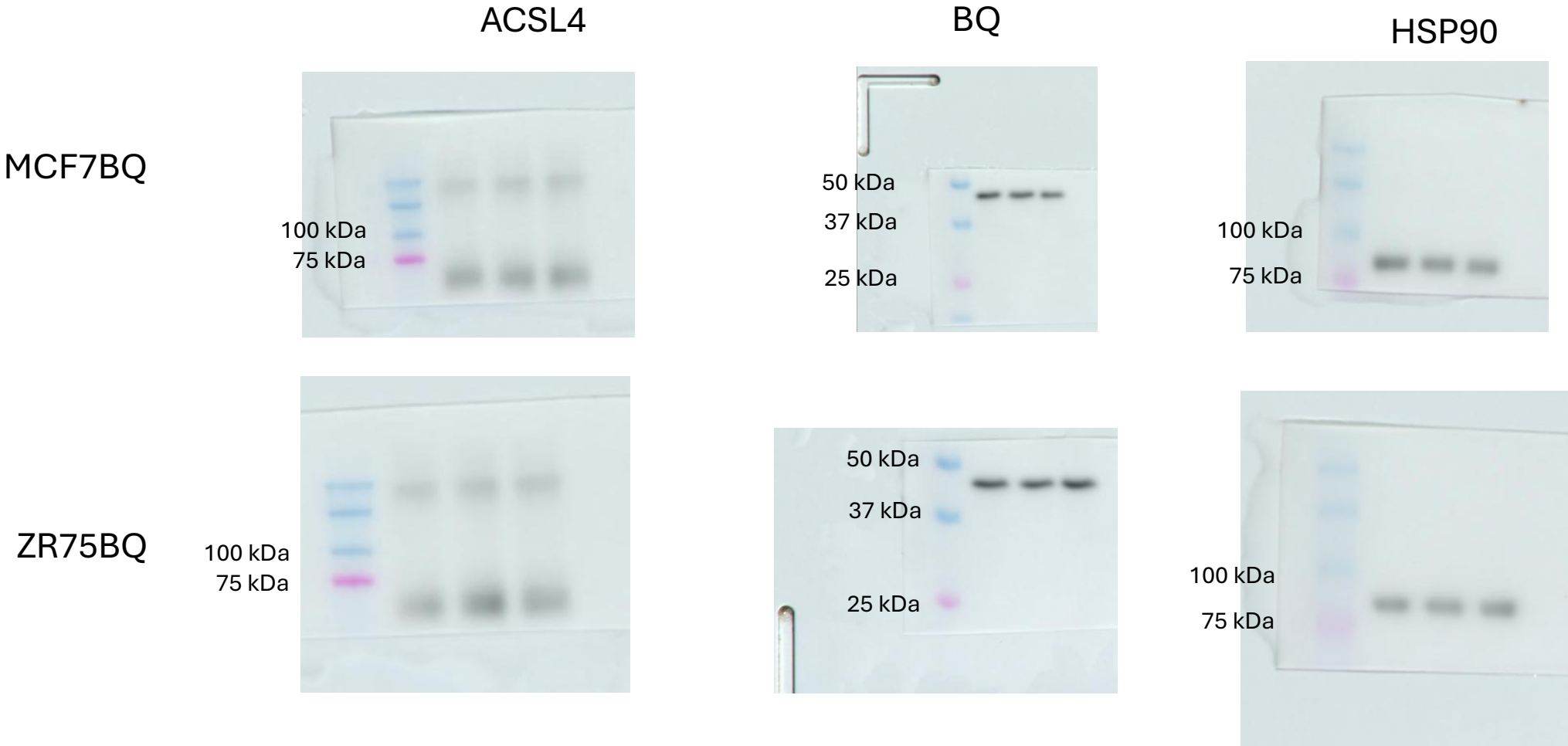

Figure S3

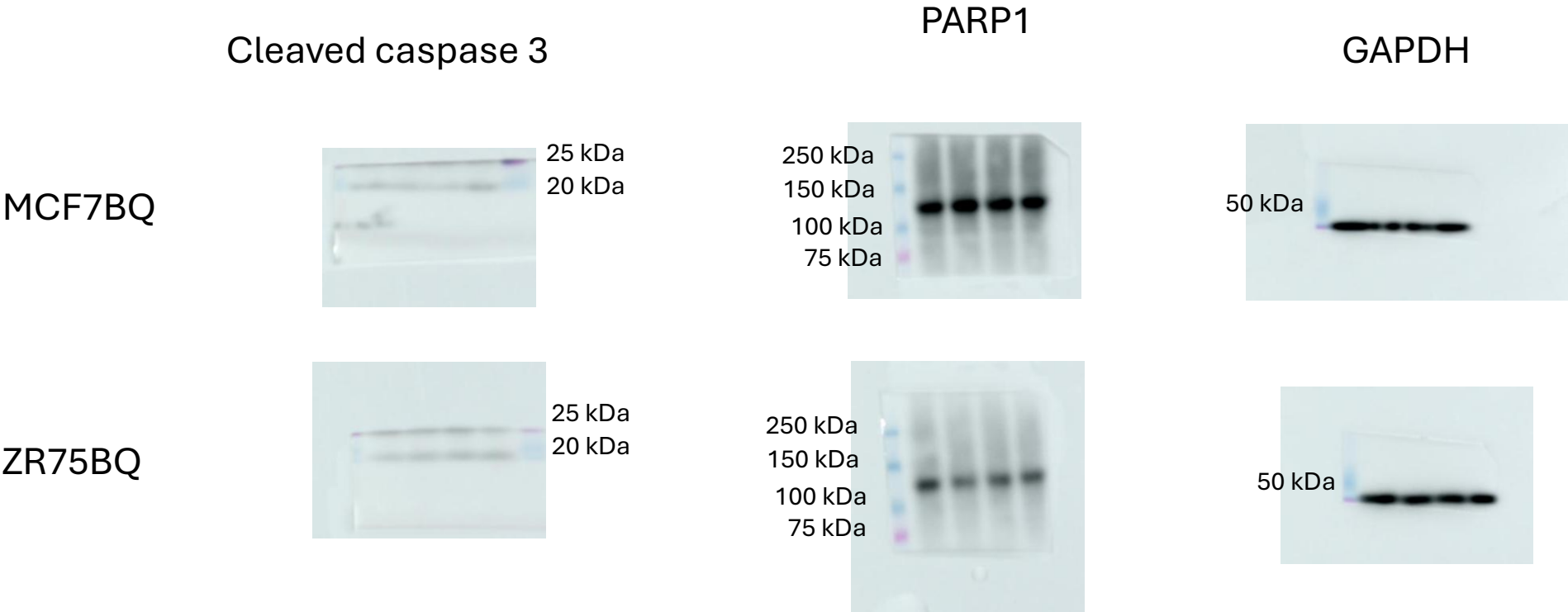

Supplement: Supplementary file 1 [file ijms-26-04989-s001.zip › 20250516_ACSL4 manuscript_raw blot_revision.pdf]

Figure S1

A

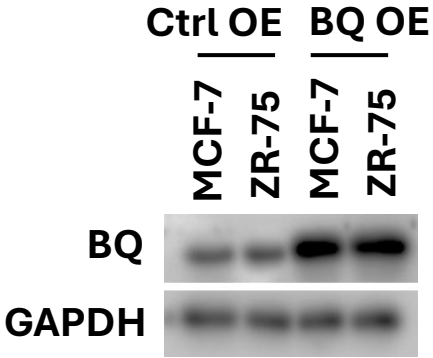

B

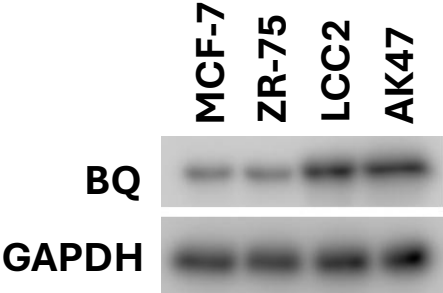

C

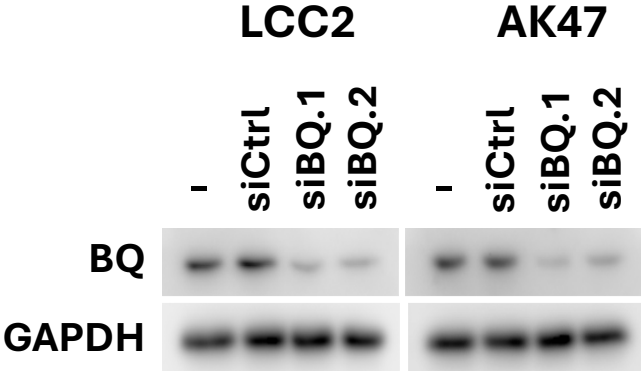

Figure S2

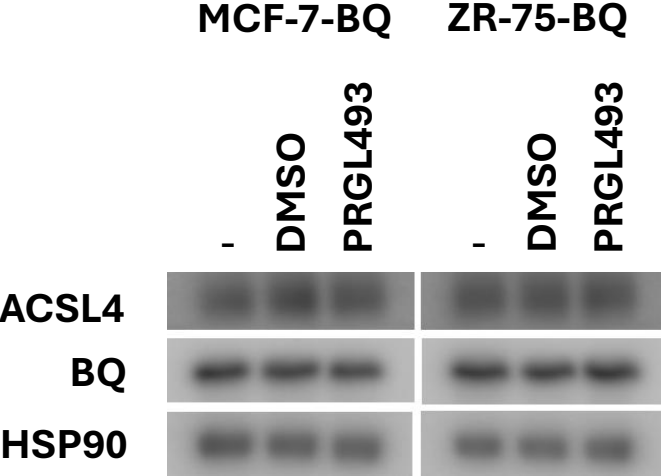

Figure S3

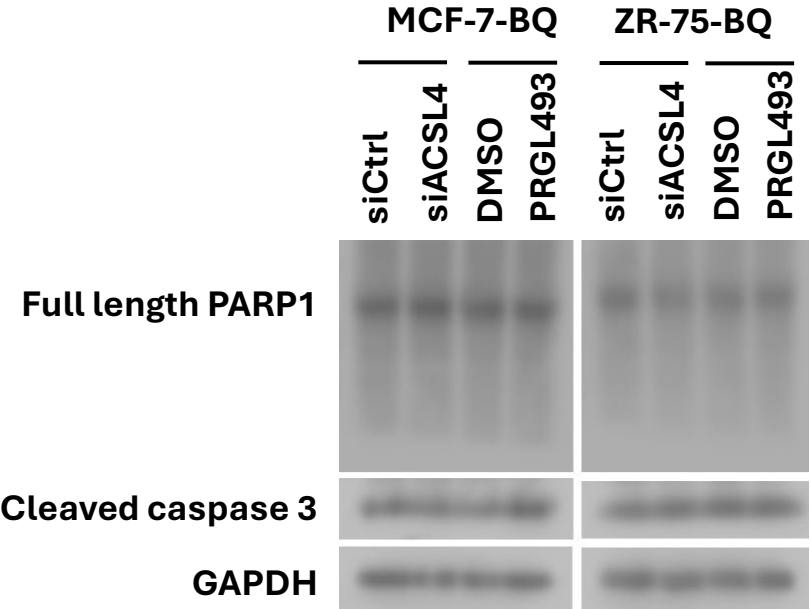

Figure S4

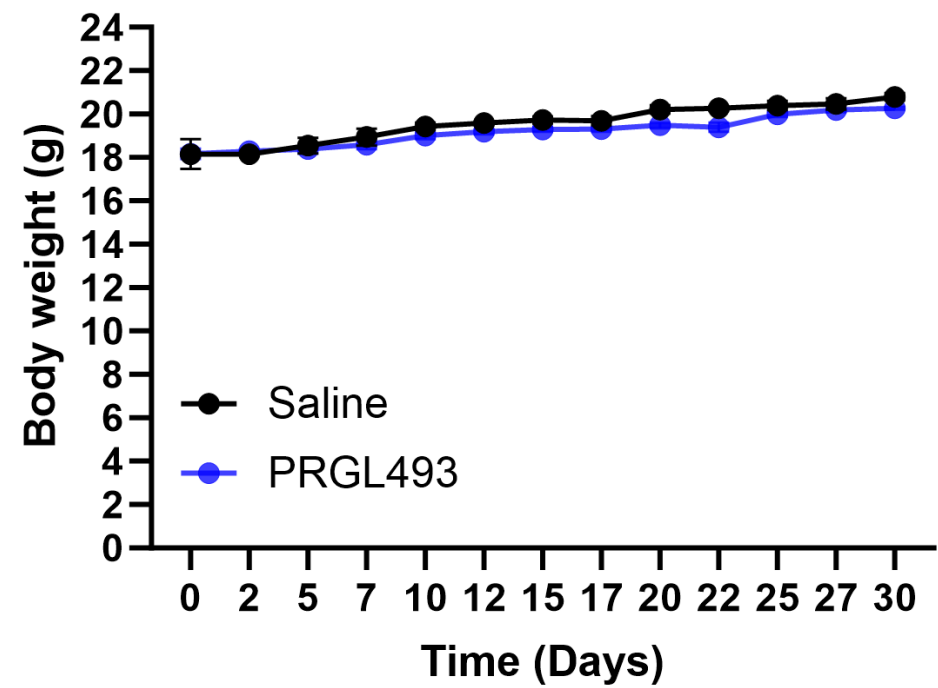

Supplement: Supplementary file 1 [file ijms-26-04989-s001.zip › 20250516_ACSL4_supp figure_revision.pdf]
